# Association between the atherogenic index of plasma and mortality in the chronic kidney disease population: evidence from NHANES

**DOI:** 10.3389/fmed.2025.1575657

**Published:** 2025-05-30

**Authors:** Luohua Li, Jinhan Zhao, Mei Yuan, Hongying Jiang

**Affiliations:** ^1^Department of Nephrology, The Second Hospital Affiliated to Kunming Medical University, Kunming, China; ^2^Department of Nephrology, Jiujiang City Key Laboratory of Cell Therapy, Jiujiang No. 1 People’s Hospital, Jiujiang, China; ^3^The Third Unit of the Department of Hepatology, Beijing Institute of Hepatology, Beijing Youan Hospital, Capital Medical University, Beijing, China

**Keywords:** atherogenic index of plasma, mortality, National Health and Nutrition Examination Survey, chronic kidney disease, cardiovascular disease, prognosis

## Abstract

**Background:**

The atherosclerosis index (AIP) in plasma is a novel indicator closely associated with various metabolic abnormalities, and the bidirectional relationship between metabolic dysfunction and chronic kidney disease (CKD) has been extensively documented. However, evidence regarding the association between AIP and mortality in CKD population remains scarce. This study aims to elucidate the association between baseline AIP levels and both all-cause and specific mortality in a diverse cohort of US adults.

**Methods:**

This cohort study utilized data from the National Health and Nutrition Examination Survey (NHANES) spanning from 1999 to 2018. A total of 4,403 participants were included in the analysis. Mortality rates were determined through linkage with the National Death Index (NDI), with follow-up extending through December 31, 2019. The primary outcome variables were all-cause mortality and cause-specific mortality. Multivariable weighted Cox proportional hazards regression models, restricted cubic spline analysis, subgroup stratification, and sensitivity testing were utilized to evaluate the associations between the AIP and both all-cause and cause-specific mortality.

**Results:**

During a median follow-up of 83 months, 1,767 all-cause deaths and 526 CVD deaths occurred. After multivariable adjustment, AIP was independently associated with elevated risks of all-cause mortality (HR: 2.03, 95% CI: 1.62∼2.55, *P* < 0.001) and cardiovascular mortality (SHR: 1.60, 95% CI: 1.08∼2.39, *P* = 0.028). Restricted cubic spline analysis confirmed a linear dose-response relationship between AIP and all-cause mortality (*P* for non-linearity = 0.243). Subgroup analyses confirmed consistent associations across demographics and comorbidities, with significant interactions observed for sex, BMI, and diabetes (*P* < 0.05). Sensitivity analyses excluding deaths within 2 years showed similar outcomes (all-cause mortality HR: 1.94, 95% CI: 1.53∼2.47, *P* < 0.001; CVD mortality SHR: 1.69, 95% CI: 1.13∼2.53, *P* = 0.01).

**Conclusion:**

Data from large cohort studies have revealed a significant positive correlation between AIP levels and the risks of all-cause and cardiovascular mortality in the adult population of the United States. This suggests that AIP is associated with an increased risk of adverse outcomes in CKD and may serve as biomarker.

## 1 Introduction

Chronic kidney disease (CKD), as a major global public health concern, is receiving increasingly extensive attention. Over the past several decades, its incidence and prevalence have manifested a notable upward trend. According to relevant estimations, approximately 700 million people worldwide are afflicted by CKD, which results in approximately 1.2 million deaths and directly contributes to 35.8 million disability-adjusted life years (DALYs) (1, 2). The prevalence of CKD varies among different regions. In low- and middle-income countries, the prevalence is relatively high, even affecting more than 10% of the local population (3). Taking the United States as an example, the estimated prevalence of CKD is approximately 15%, and in 2017 alone, medical expenditures related to CKD management exceeded 84 billion US dollars (4, 5). Beyond its direct impact on renal function, CKD is intricately linked to cardiovascular complications (6–8). This cardiovascular burden is further exacerbated by metabolic derangements, particularly dyslipidemia, which accelerates glomerular injury and systemic inflammation (9).

In recent years, a considerable number of studies have indicated that lipid metabolism disorders exert a critical role in the pathogenesis and progression of CKD (6, 9–11). Atherosclerosis index (AIP), a novel lipid marker initially proposed by Dobiasova and Frohlich in 2001, is obtained through logarithmic transformation of the ratio of triglycerides (TG) to high-density lipoprotein cholesterol (HDL-C) (12). AIP is capable of reflecting the characteristics and extent of lipid metabolism abnormalities. A growing body of research evidence highlights the significance of AIP in predicting the risk of cardiovascular disease (13, 14), as well as metabolic disorders such as non-alcoholic fatty liver disease (NAFLD) (15). Nonetheless, its role in CKD remains insufficiently explored. Especially, a sufficient and in-depth cognition of the consistency and specificity of this association among different regional populations has not yet been formed. Cross-sectional analyses have associated AIP with albuminuria and reduced glomerular filtration rate (16–18), yet longitudinal data are lacking. Mechanistically, AIP may drive renal damage through endothelial dysfunction, oxidative stress, and proinflammatory pathways (5). Recent trials demonstrate that sodium-glucose cotransporter 2 (SGLT2) inhibitors reduce cardiovascular mortality in CKD patients by modulating lipid metabolism and mitigating inflammation (2, 5). However, these therapies do not address the fundamental dyslipidemia inherent to CKD, underscoring the need for novel biomarkers like AIP. Hence, an in-depth exploration of the specific role of AIP in the CKD population and its impact on prognosis is of paramount significance for optimizing the management strategies for CKD patients.

The present study sought to investigate the longitudinal association between baseline AIP levels and mortality outcomes in a nationally representative CKD cohort.

## 2 Material and methods

### 2.1 Study population

**FIGURE 1:**
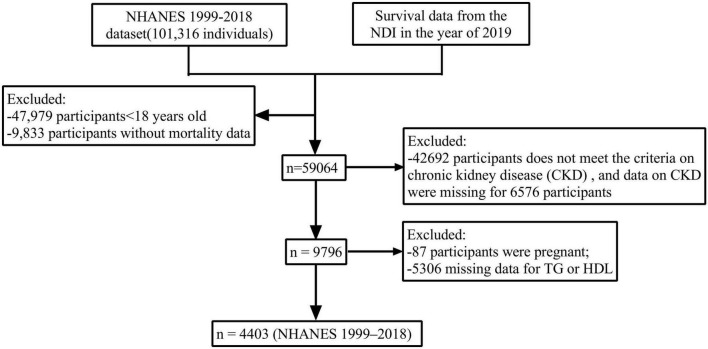
Flow chart of participant selection from the NHANES 1999–2018 dataset. A total of 4,403 adults with chronic kidney disease (CKD) were included in the final analysis. Exclusion criteria included age < 18 years, missing mortality data, incomplete CKD diagnostic criteria, pregnancy, and missing TG/HDL-C measurements.

NHANES is an ongoing periodic cross-sectional sample survey conducted by the National Center for Health Statistics (NCHS) within the U.S. Centers for Disease Control and Prevention (CDC). This survey aims to collect data from a nationally representative sample of the non-institutionalized civilian population. Mortality data are sourced from the NDI database maintained by the CDC. This study analyzed data from all 11 consecutive survey cycles of NHANES conducted between 1999 and 2018, focusing specifically on non-pregnant adults aged 18 years and older, while excluding individuals with incomplete data. Participants were followed until their death or until December 31, 2019. Ultimately, 4,403 eligible participants were included in the analysis to investigate the relationship between AIP and all-cause mortality within the CKD population (Figure 1).

For details on survey design and data acquisition, please refer to the NHANES website at https://www.cdc.gov/nchs/nhanes/ (accessed January 5, 2025). The NHANES research plan has received approval from the NCHS Ethics Review Board, as outlined at https://www.cdc.gov/nchs/nhanes/about/erb.html?CDC_AA_refVal=https://www.cdc.gov/nchs/nhanes/irba98.htm. Furthermore, All participants provided written informed consent for the use of their data, and all experiments conducted in this study adhered to relevant guidelines and regulations.

CKD can be defined by two primary criteria: firstly, a glomerular filtration rate (GFR) of less than 60 mL/(min⋅1.73 m^2^); and secondly, the presence of one or more indicators of kidney damage, which include: (a) albuminuria, defined as a urine albumin-to-creatinine ratio (ACR) of ≥ 30 mg/g; (b) abnormal urinary sediment; (c) electrolyte or other abnormalities resulting from renal tubular dysfunction; (d) renal histological abnormalities; and (e) renal structural abnormalities identified through imaging examinations. A diagnosis of CKD can be made if any of these conditions persist for at least 3 months (19). The severity of CKD is categorized into five stages based on the estimated glomerular filtration rate (eGFR): G1 (eGFR ≥ 90 mL/min/1.73 m^2^), G2 (eGFR 60–89 mL/min/1.73 m^2^), G3 (eGFR 30–59 mL/min/1.73 m^2^), G4 (eGFR 15–29 mL/min/1.73 m^2^), and G5 (eGFR < 15 mL/min/1.73 m^2^). Each stage reflects varying degrees of renal function impairment (20).

### 2.2 Study variables

#### 2.2.1 Exposure variable

The calculation formula for the AIP is expressed as follows: AIP = Log [TG (mmol/L)/HDL-C (mmol/L)] (21). This indicator is derived from peripheral blood samples collected after a fasting period of at least 8 hours in the morning, during which TG and HDL-C levels were measured. Following the standardized protocol established by the CDC, serum HDL-C levels were assessed using either a direct immunoassay or a precipitation method, while serum TG levels were measured using enzymatic methods (22). Based on the quartiles of AIP, the study population was categorized into four groups: Q1 group (≤ −0.19), Q2 group (−0.19 to 0.02), Q3 group (0.02–0.24), and Q4 group (> 0.24), for subsequent analysis of the association between AIP and mortality.

#### 2.2.2 Covariates

The included demographic characteristics encompass age, gender, race, family poverty-income ratio (FPIR), marital status, body mass index (BMI), education level (categorized as below high school, high school graduate or equivalent, and above high school), drinking habits (classified as never, former, mild, moderate, and severe), and smoking status (comprising never smokers, former smokers, and current smokers). Laboratory test data consist of white blood cell count (WBC), neutrophils, hemoglobin, creatinine, uric acid, blood urea nitrogen, TG, total cholesterol (TC), HDL-c, low-density lipoprotein cholesterol (LDL-c), C-reactive protein (CRP), estimated glomerular filtration rate (eGFR), and systemic immune inflammatory index (SII). Additionally, chronic comorbidities such as diabetes, hypertension, cardiovascular disease, hyperlipidemia, and the use of lipid-lowering medications were documented. The household poverty-income ratio is determined by comparing total household income to the poverty line. Smoking status is classified into three categories: current smokers (more than 100 cigarettes in lifetime and currently smoking all or some days), former smokers (having smoked more than 100 cigarettes throughout life but having quit currently), and never smokers (having smoked less than 100 cigarettes throughout life).

### 2.3 Mortality outcome of the study population

The mortality data for this study were obtained from the NDI as of December 31, 2019. The database utilized the 10th revision of the International Classification of Diseases and Related Health Problems (ICD-10) to record the leading causes of death. The aim of this study was to investigate the effect of AIP levels on all-cause and cardiovascular mortality. All-cause mortality refers to death from any cause during the follow-up period. Cardiovascular mortality was identified using the International Classification of Diseases, 10th Revision codes I00–I09, I11, I13, I20-I51, or I60–I69. Deaths due to causes such as infection (ICD-10: J09-J18, or J40-J47) and cancer (CA) (ICD-10: C00-C97) were analyzed as additional competing outcomes (23, 24).

### 2.4 Statistical analysis

Given that the NHANES database employs a complex, multistage, stratified sampling design, we adhered to the guidelines established by the CDC and utilized appropriate weighting methods to address this complexity. All analyses were weighted to account for issues arising from complex survey designs, ensuring that the estimates were representative of the general population (19). For continuous variables, we report the weighted mean ± standard error (SE) and median (interquartile range), while for categorical variables, we present estimated proportions. We employed analysis of variance (ANOVA) or the rank sum test to evaluate group differences for continuous variables, and the chi-square test for categorical variables. To compare survival estimates and cumulative incidence rates, we employed both the Kaplan-Meier method and competing risk models. After confirming the proportional hazards assumption for the influence of AIP on survival risk through the Schoenfeld residual test, we conducted multivariable Cox proportional hazards regression analyses to assess the association between AIP levels and all-cause mortality. The results were expressed as hazard ratios (HRs) with corresponding 95% confidence intervals (CIs). Additionally, we implemented the Fine-Gray competing risk regression model to examine the relationship between AIP levels and CVD mortality, presenting the findings as subdistribution hazard ratios (SHRs) with 95% CIs. Furthermore, a *post hoc* power analysis, based on observed event rates and effect sizes, confirmed that our sample size was sufficient to detect significant associations (α = 0.05, power = 80%).

Analytical models were refined incrementally to address potential confounding factors. The Crude Model did not adjust for any covariates, while Model 1 included adjustments for gender, age, and race. Model 2 further adjusted for smoking status, drinking status, education levels, marital status, FPIR, hypertension, diabetes, history of cardiovascular disease, history of lipid-lowering drug use, CRP, SII, LDL-c, eGFR, and additional covariates from Model 1. Using patients in the lowest weighted quartile of AIP as the reference group, we calculated the HR, 95% CI, and *P* for trend regarding the risk of all-cause mortality, as well as the SHR, 95% CI, and *P* for trend for CVD mortality. Restricted cubic spline plots were employed to illustrate the association between AIP as a continuous variable and all-cause mortality, while likelihood ratio tests were utilized to assess potential non-linear relationships. Subgroup analyses were conducted based on age, gender, BMI, race, FPIR, hypertension, diabetes, and education level. To address potential reverse causality, we conducted sensitivity analyses excluding deaths within the initial two follow-up years. Prognostic performance was assessed through the concordance index (C-index), with incremental value quantified using Integrated Discrimination Improvement (IDI) and Net Reclassification Improvement (NRI) metrics relative to established risk factors. Statistical analyses were performed using R statistical software (version 4.3.2; R Foundation for Statistical Computing, Vienna, Austria), with two-sided *P*-values < 0.05 considered statistically significant.

## 3 Results

### 3.1 Baseline characteristics of study participants

A total of 4,403 patients diagnosed with CKD were encompassed in this study. The weighted median age of the participants was 63.3 years, with 53.0% being female. Table 1 presents the baseline characteristics of the patients in accordance with the weighted quartiles of AIP. The weighted median of AIP was 0.20 (interquartile range: −0.19 to 0.24). The study findings demonstrated that patients with elevated AIP values generally exhibited higher body mass index (BMI), white blood cell count, neutrophil count, C-reactive protein level, systemic inflammation index (SII), uric acid and low-density lipoprotein cholesterol (LDL), while having lower estimated glomerular filtration rate (eGFR). Furthermore, such patients were more likely to have lower educational attainment, be married or cohabiting with a partner, mainly distributed in the Mexican American and non-Hispanic white populations, and had a higher prevalence of hypertension, diabetes, and cardiovascular diseases (including coronary heart disease). In contrast, patients with lower AIP values tended to be younger.

**TABLE 1:** Baseline characteristics of patients stratified by AIP quartiles.

AIP	Total (unweighted *n* = 4,403)	Quartile 1 (≤ −0.19) (unweighted *n* = 1,101)	Quartile 2 (−0.19 to 0.02) (unweighted *n* = 1,100)	Quartile 3 (0.02–0.24) (unweighted *n* = 1,101)	Quartile 4 (>0.24) (unweighted *n* = 1101)	*P*-value
Weighted, n	13,171,333	3,401,221	3,167,630	3,187,506	3,414,975	–
Age (years)	63.3 ± 17.8	61.4 ± 20.2	64.4 ± 18.2	65.0 ± 16.2	62.4 ± 15.9	<0.001
Gender, n (%)						<0.001
Female	2335 (53.0)	697 (63.3)	628 (57.1)	557 (50.6)	453 (41.1)	
Male	2068 (47.0)	404 (36.7)	472 (42.9)	544 (49.4)	648 (58.9)	
BMI, kg/m^2^	29.7 ± 7.4	26.8 ± 6.9	29.0 ± 7.6	31.3 ± 7.4	31.5 ± 6.6	<0.001
Smoking status, n (%)						<0.001
Former	1423 (33.0)	310 (29.3)	317 (29.5)	394 (36.1)	402 (36.8)	
Never	2175 (50.4)	574 (54.3)	589 (54.8)	526 (48.3)	486 (44.5)	
Now	716 (16.6)	174 (16.4)	169 (15.7)	170 (15.6)	203 (18.6)	
Drinking status (%)						<0.001
Never	698 (18.2)	160 (17.1)	170 (18.1)	188 (19.4)	180 (18.1)	
Former	1046 (27.2)	216 (23)	235 (25)	291 (30.1)	304 (30.5)	
Mild	1249 (32.5)	312 (33.3)	327 (34.8)	295 (30.5)	315 (31.6)	
Moderate	363 (9.4)	117 (12.5)	99 (10.5)	73 (7.5)	74 (7.4)	
Heavy	487 (12.7)	133 (14.2)	110 (11.7)	120 (12.4)	124 (12.4)	
Race, n (%)						<0.001
Mexican American	718 (16.3)	108 (9.8)	170 (15.5)	196 (17.8)	244 (22.2)	
Non-Hispanic Black	932 (21.2)	363 (33)	258 (23.5)	189 (17.2)	122 (11.1)	
Non-Hispanic White	2141 (48.6)	486 (44.1)	526 (47.8)	545 (49.5)	584 (53)	
Other Hispanic	304 (6.9)	60 (5.4)	73 (6.6)	86 (7.8)	85 (7.7)	
Other Race - Including Multi-Racial	308 (7.0)	84 (7.6)	73 (6.6)	85 (7.7)	66 (6)	
Marry status, n (%)						<0.001
Divorced/Widowed/ Separated	1541 (35.7)	409 (38.3)	409 (37.8)	398 (36.6)	325 (30.1)	
Married/Living with partner	2314 (53.6)	501 (47)	552 (51)	598 (55)	663 (61.3)	
Never married	462 (10.7)	157 (14.7)	121 (11.2)	91 (8.4)	93 (8.6)	
Education levels, n (%)						<0.001
Below high school	800 (18.2)	135 (12.3)	201 (18.3)	220 (20.1)	244 (22.2)	
High school	1835 (41.8)	458 (41.7)	454 (41.4)	481 (43.9)	442 (40.1)	
Over high school	1755 (40.0)	505 (46)	441 (40.2)	394 (36)	415 (37.7)	
FPIR, n (%)						0.237
<1	917 (23.1)	233 (23.5)	223 (22.4)	217 (22)	244 (24.3)	
1–3	1900 (47.8)	445 (44.9)	489 (49.1)	473 (48)	493 (49)	
≥3	1159 (29.1)	312 (31.5)	283 (28.4)	295 (29.9)	269 (26.7)	
Hypertension, n (%)						<0.001
No	1262 (28.7)	389 (35.3)	318 (28.9)	287 (26.1)	268 (24.3)	
Yes	3140 (71.3)	712 (64.7)	782 (71.1)	813 (73.9)	833 (75.7)	
Diabetes, n (%)						<0.001
No	2581 (58.6)	809 (73.5)	689 (62.6)	596 (54.1)	487 (44.2)	
Yes	1822 (41.4)	292 (26.5)	411 (37.4)	505 (45.9)	614 (55.8)	
CVD, n (%)						<0.001
No	3055 (71.2)	782 (75)	778 (72.7)	737 (67.6)	758 (69.5)	
Yes	1238 (28.8)	261 (25)	292 (27.3)	353 (32.4)	332 (30.5)	
Hyperlipidemia, n (%)						<0.001
No	795 (18.1)	403 (36.6)	289 (26.3)	103 (9.4)	0 (0)	
Yes	3608 (81.9)	698 (63.4)	811 (73.7)	998 (90.6)	1101 (100)	
Lipid lowering drugs, n (%)						<0.001
No	888 (20.2)	274 (24.9)	221 (20.1)	206 (18.7)	187 (17)	
Yes	1550 (35.2)	340 (30.9)	378 (34.4)	447 (40.7)	385 (35)	
WBC (10^9^/L)	7.2 ± 3.3	6.5 ± 2.2	7.1 ± 4.0	7.4 ± 2.5	7.7 ± 3.8	<0.001
Neutrophil (10^9^/L)	4.4 ± 2.1	3.9 ± 1.9	4.3 ± 1.7	4.5 ± 1.6	4.7 ± 2.9	<0.001
Hemoglobin (g/L)	13.8 ± 1.7	13.4 ± 1.7	13.6 ± 1.7	13.9 ± 1.7	14.3 ± 1.7	<0.001
Creatinine (μmol/L)	88.4 (70.7, 114.9)	85.8 (65.4, 107.0)	88.4 (70.7, 114.9)	92.8 (70.7, 116.7)	88.4 (70.7, 114.9)	<0.001
Uric acid (μmol/L)	360.0 ± 100.0	330.1 ± 91.9	354.3 ± 96.2	371.0 ± 102.6	384.5 ± 100.9	<0.001
Blood urea (mmol/L)	5.7 (4.3, 7.8)	5.7 (3.9, 7.5)	6.1 (4.3, 7.8)	6.1 (4.3, 8.2)	5.7 (4.3, 7.8)	<0.001
TG (mmol/L)	1.7 ± 1.6	0.8 ± 0.2	1.2 ± 0.3	1.6 ± 0.4	3.2 ± 2.5	<0.001
TC (mmol/L)	5.0 ± 1.2	4.8 ± 1.1	4.9 ± 1.2	4.9 ± 1.1	5.4 ± 1.4	<0.001
HDL-c (mmol/L)	1.4 ± 0.4	1.8 ± 0.5	1.4 ± 0.3	1.2 ± 0.2	1.0 ± 0.2	<0.001
LDL-c (mmol/L)	2.9 ± 1.0	2.6 ± 0.9	2.9 ± 1.0	2.9 ± 1.0	3.0 ± 1.1	<0.001
CRP (mg/L)	0.3 (0.1, 0.7)	0.2 (0.1, 0.4)	0.3 (0.1, 0.7)	0.3 (0.1, 0.7)	0.4 (0.2, 0.8)	<0.001
eGFR[mL⋅min^–1^⋅(1.73 m^2^)^–1^]	72.1 ± 30.2	76.4 ± 31.4	71.0 ± 30.4	69.2 ± 29.4	71.9 ± 29.2	<0.001
SII	522.0 (360.0, 755.9)	483.5 (326.2, 713.0)	525.0 (375.5, 768.2)	537.8 (374.5, 776.1)	536.2 (372.1, 793.1)	<0.001

Values are weighted mean ± SE for continuous variables or weighted% for categorical variables. Abbreviations: AIP, atherogenic index of plasma; BMI, body mass index; FPIR, family poverty-income ratio; CVD, cardiovascular disease; WBC, white blood cell; TG, Triglyceride; TC, total Cholesterol; LDL-c, low-density lipoprotein; eGFR, estimated glomerular filtration rate; CRP, C-reaction protein; SII, systemic immune-inflammation index.

### 3.2 AIP and mortality in CKD participants

**FIGURE 2:**
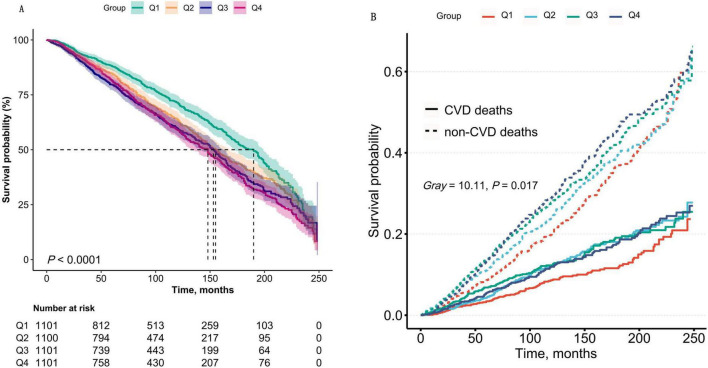
**(A)** Kaplan–Meier survival curves for all-cause mortality stratified by AIP quartiles (Q1–Q4) in CKD patients. The log-rank test demonstrated a significant difference in survival across quartiles (*P* < 0.0001). Q1: AIP ≤ -0.19; Q2: - 0.19 to 0.02; Q3: 0.02–0.24; Q4: > 0.24. **(B)** Cumulative incidence of CVD mortality analyzed using Fine-Gray competing risk models, accounting for non-CVD deaths as competing events. The Gray’s test confirmed significant differences in CVD mortality risk across AIP quartiles (*P* < 0.0001). Shaded areas represent 95% confidence intervals. Data were weighted to represent the U.S. population.

During a median follow-up period of 83 months (interquartile range: 43–137 months), a total of 416,840 person-months of observation data were collected. During this period, 1,767 patients with CKD succumbed to all-cause mortality, including 526 deaths attributable to CVD, 305 due to malignant neoplasms, and 109 resulting from infectious diseases. Results from Kaplan-Meier survival analysis and Fine-Gray competing risk models demonstrated that CKD patients in the highest quartile (Q4) of the AIP exhibited significantly elevated risks of both all-cause mortality and CVD mortality compared to those in lower AIP groups (*P* < 0.05), as illustrated in Figure 2. Furthermore, Fine-Gray competing risk analysis revealed no significant differences in CA-related or infection-related mortality between CKD patients in the Q4 and those in the lower quartiles (*P* > 0.05), as detailed in Supplementary Figure 1.

Table 2 presents detailed hazard ratios (HRs) and 95% CIs for the association between the AIP and all-cause mortality, as well as subdistribution hazard ratios (SHRs) and 95% CIs for cardiovascular mortality, including both unadjusted and multivariable-adjusted analyses. After adjusting for sociodemographic factors, laboratory parameters, health-related lifestyle behaviors, and comorbidities, AIP was found to be significantly associated with an increased risk of all-cause mortality in CKD patients (HR: 2.03, 95% CI: 1.62–2.55, *P* < 0.001) (Table 2). Additionally, the results indicate a significant positive association between AIP and cardiovascular mortality risk (HR: 1.60, 95% CI: 1.08–2.39, *P* = 0.02). These findings suggest that each unit increase in AIP corresponds to a 103% higher risk of all-cause mortality and a 60% higher risk of cardiovascular mortality among CKD patients.

**TABLE 2:** Association between AIP and all-cause mortality (Cox regression model), and AIP and CVD mortality (Fine-Gray competing risk model).

Outcome	Crude model		Model 1		Model 2	
**All-cause mortality**	**HR (95% CI)**	***P-*value**	**HR (95% CI)**	***P*-value**	**HR (95% CI)**	***P*-value**
AIP	1.45 (1.27∼1.65)	<0.001	1.67 (1.44∼1.92)	<0.001	2.03 (1.62∼2.55)	<0.001
**AIP quartile**						
Quartile 1	Reference		Reference		Reference	
Quartile 2	1.27 (1.11∼1.46)	0.001	1.23 (1.07∼1.41)	0.004	1.29 (1.04∼1.6)	0.02
Quartile 3	1.42 (1.24∼1.63)	<0.001	1.41 (1.23∼1.62)	<0.001	1.42 (1.14∼1.77)	0.002
Quartile 4	1.46 (1.28∼1.67)	<0.001	1.52 (1.32∼1.75)	<0.001	1.90 (1.53∼2.38)	<0.001
*P* for trend	<0.001		<0.001		<0.001	
**CVD mortality**	**SHR (95% CI)**	***P*-value**	**SHR (95% CI)**	***P*-value**	**SHR (95% CI)**	***P*-value**
AIP	1.57 (1.14∼2.16)	0.006	1.49 (1.16∼1.91)	0.002	1.60 (1.08∼2.39)	0.02
**AIP quartile**						
Quartile 1	Reference		Reference		Reference	
Quartile 2	1.81 (1.22∼2.7)	0.003	1.40 (1.09∼1.79)	0.008	1.54 (1.04∼2.30)	0.033
Quartile 3	1.64 (1.09∼2.49)	0.019	1.45 (1.13∼1.88)	0.004	1.41 (0.92∼2.15)	0.114
Quartile 4	1.94 (1.30∼2.89)	0.001	1.58 (1.22∼2.05)	0.001	1.73 (1.13∼2.63)	0.011
*P* for trend	0.003		0.001		0.028	

Crude Model: unadjusted. Model 1: adjust for gender, age, Race. Model 2: adjusted for Model 1 + smoking status, drinking status, education levels, marriage status, FPIR, hypertension, diabetes, CVD history, Lipid lowering drugs history, CRP, SII, LDL-c, eGFR.

When analyzed as a categorical variable, the results are summarized in Table 2. Across the ascending AIP categories (0 to −0.19, >−0.19 to 0.02, > 0.02–0.24, and > 0.24), the weighted estimated hazard ratios (HRs, 95% CIs) for all-cause mortality in CKD patients demonstrated a significant dose-response relationship (P for trend < 0.001). Similarly, the weighted competing risk regression analysis indicated a progressively increasing risk of cardiovascular mortality after multivariable adjustment (P for trend < 0.001). These findings provide robust evidence that elevated AIP is significantly associated with higher mortality risk in CKD patients (*P* < 0.001).

### 3.3 The relationship between lead concentration and CKD mortality

**FIGURE 3:**
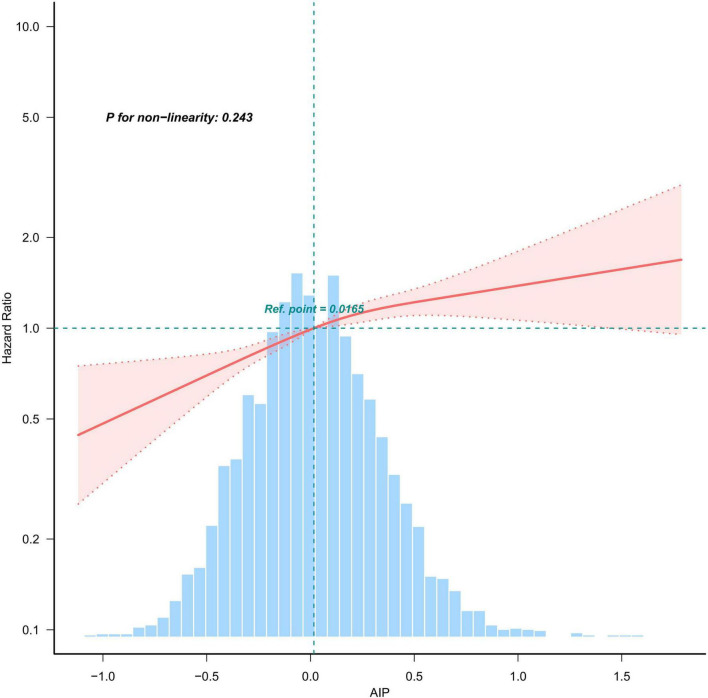
Weighted restricted cubic spline plot showing the linear relationship between AIP (continuous) and all-cause mortality with CKD. The weighted restricted cubic spline model was adjusted for the variables listed in the fully adjusted model in Table 2. The solid red line represents the HR derived from the weighted multivariable Cox model, while shadows represent corresponding 95% CIs of the adjusted HR. The blue histogram illustrates the frequency distribution across various intervals of AIP content.

A multivariate-adjusted weighted restricted cubic spline analysis revealed a significant linear relationship between AIP and the risk of all-cause mortality (P for non-linearity = 0.243). This finding indicates that higher AIP levels are associated with an increased risk of all-cause mortality, as shown in Figure 3.

### 3.4 Subgroup analysis

This study performed a subgroup analysis to investigate the relationships between AIP values and the risks of all-cause and cardiovascular mortality across various demographic characteristics and comorbid conditions, as detailed in Table 3. Stratifications were conducted by age (< 60 years vs. ≥ 60 years), sex (male vs. female), body mass index (BMI; < 25 kg/m^2^, 25–30 kg/m^2^, ≥ 30 kg/m^2^), FPIR (< 1, 1–3, ≥ 3), race, educational level, hypertension, and diabetes mellitus (DM). The analysis revealed that the association between AIP and cardiovascular mortality was consistently observed across all subgroups (all P for interaction > 0.05). After adjusting for confounding factors, the subgroup findings remained broadly consistent with the primary results. Notably, sex, BMI, and DM were identified as significant interaction factors influencing the relationship between AIP and all-cause mortality (P for interaction < 0.05). Further details are provided in Table 3.

**TABLE 3:** Subgroup analysis of the associations between AIP and mortality among CKD.

Characteristics	All-cause mortality		*P* for interaction	CVD mortality		*P* for interaction
	HR (95% CI)	*P*-value		SHR (95% CI)	*P*-value	
**Gender**						
Female	2.41 (1.71∼3.4)	<0.001	0.003	1.57 (1.04∼2.36)	0.031	0.706
Male	1.39 (1.09∼1.78)	0.009		1.6 (1.14∼2.25)	0.007	
**Age, years**						
<65	2.35 (1.61∼3.44)	<0.001	0.172	2.46 (1.46∼4.16)	0.001	0.105
≥65	1.98 (1.55∼2.54)	<0.001		1.48 (1.10∼2.00)	0.01	
**BMI, kg/m^2^**						
<25	3.42 (2.28∼5.12)	< 0.001	0.012	1.55 (0.86∼2.80)	0.147	0.915
25–30	1.56 (1.11∼2.17)	0.009		1.47 (0.92∼2.35)	0.111	
≥30	1.70 (1.17∼2.45)	0.005		1.7 (1.09∼2.64)	0.019	
**Race**						
Mexican American	2.79 (1.63∼4.78)	< 0.001	0.072	2.14 (0.88∼5.20)	0.091	0.483
Non-Hispanic Black	3.26 (1.83∼5.79)	< 0.001		2.88 (1.60∼5.19)	< 0.001	
Non-Hispanic white	1.53 (1.20∼1.95)	0.001		1.72 (1.23∼2.41)	0.001	
Other Hispanic	1.34 (0.44∼4.08)	0.611		0.85 (0.18∼3.92)	0.835	
Other Race	1.68 (0.48∼5.85)	0.414		1.48 (0.30∼7.36)	0.635	
**FPIR**						
<1	1.53 (0.96∼2.43)	0.072	0.546	1.56 (0.82∼2.94)	0.174	0.346
1–3	2.11 (1.54∼2.89)	< 0.001		1.48 (1.02∼2.15)	0.041	
≥3	1.72 (1.20∼2.46)	0.003		2.18 (1.30∼3.67)	0.003	
**Hypertension**						
No	1.9 (1.27∼2.85)	0.002	0.503	1.23 (0.65∼2.33)	0.529	0.262
Yes	1.64 (1.30∼2.06)	< 0.001		1.73 (1.29∼2.31)	< 0.001	
**DM**						
No	2.51 (1.87∼3.37)	< 0.001	<0.001	1.34 (0.93∼1.94)	0.119	0.386
Yes	1.10 (0.82∼1.49)	0.515		1.75 (1.18∼2.59)	0.006	
**Education levels**						
Below high school	2.09 (1.36∼3.19)	0.001	0.775	2.06 (1.10∼3.84)	0.023	0.806
High school	1.69 (1.22∼2.35)	0.002		1.44 (0.95∼2.18)	0.086	
Over high school	1.71 (1.26∼2.32)	0.001		1.85 (1.23∼2.79)	0.003	

HRs and SHRs were adjusted for age, sex, race, BMI, smoking status, drinking status, education levels, FPIR, marriage status, hypertension, diabetes, CVD history, CRP, SII, LDL-C, and eGFR, with the exception of the stratification variable itself. Abbreviations: BMI, body mass index; FPIR, family poverty-income ratio; CVD, cardiovascular disease; LDL-c, low-density lipoprotein; eGFR, estimated glomerular filtration rate; CRP, C-reaction protein; SII, systemic immune-inflammation index.

### 3.5 Sensitivity analysis

Additionally, a sensitivity analysis excluding participants who died within the first 2 years of follow-up yielded results consistent with the primary analysis, further reinforcing the robustness of the study findings (see Table 4). These results indicate a positive association between elevated AIP levels and the risks of both all-cause and cardiovascular mortality.

**TABLE 4:** Association between AIP and all-cause mortality as well as CVD mortality, excluding participants who died within the first 2 years of follow-up (*n* = 4,176).

Outcome	Crude model		Model 1		Model 2	
**All-cause mortality**	**HR (95% CI)**	***P*-value**	**HR (95% CI)**	***P*-value**	**HR (95% CI)**	***P*-value**
AIP	1.45 (1.27∼1.67)	<0.001	1.67 (1.43∼1.95)	<0.001	1.94 (1.53∼2.47)	<0.001
**AIP quartile**						
Quartile 1	Reference		Reference		Reference	
Quartile 2	1.23 (1.06∼1.42)	0.006	1.19 (1.03∼1.38)	0.019	1.25 (1∼1.57)	0.049
Quartile 3	1.38 (1.19∼1.59)	<0.001	1.38 (1.19∼1.6)	<0.001	1.3 (1.03∼1.65)	0.028
Quartile 4	1.46 (1.27∼1.69)	<0.001	1.52 (1.31∼1.77)	<0.001	1.82 (1.44∼2.30)	<0.001
*P* for trend	<0.001		<0.001		<0.001	
**CVD mortality**	**SHR (95% CI)**	***P*-value**	**SHR (95% CI)**	***P*-value**	**SHR (95% CI)**	***P*-value**
AIP	1.58 (1.13∼2.22)	0.008	1.51 (1.16∼1.97)	0.002	1.69 (1.13∼2.53)	0.01
**AIP quartile**						
Quartile 1	Reference		Reference		Reference	
Quartile 2	1.73 (1.15∼2.61)	0.009	1.43 (1.1∼1.87)	0.007	1.54 (1.02∼2.32)	0.041
Quartile 3	1.62 (1.06∼2.48)	0.026	1.42 (1.08∼1.87)	0.012	1.5 (0.98∼2.31)	0.063
Quartile 4	1.83 (1.21∼2.77)	0.004	1.6 (1.22∼2.12)	0.001	1.72 (1.12∼2.65)	0.013
*P* for trend	0.008		0.002		0.021	

HRs and SHRs have been fully adjusted, as indicated in Model 2 of Table 2.

### 3.6 The forecast value of AIP for mortality

Based on previously identified factors (gender, age, smoking status, drinking status, hypertension, diabetes, CVD history, CRP, SII, LDL-c, eGFR) that influence all-cause mortality prognosis, as well as commonly debated risk factors for adverse CKD prognosis (education levels, marriage status, FPIR, lipid-lowering drugs history), we defined this as the traditional factors model. After incorporating the AIP model, it was renamed the traditional factors + AIP model. The statistical analysis demonstrated that AIP alone produced a C-index of 0.713 (95% CI: 0.571–0.855, *P* = 0.007) for predicting all-cause mortality. Although AIP exhibited moderate discriminative ability independently, its incremental prognostic value became more apparent when incorporated into the traditional factors model, improving the C-index from 0.811 to 0.823, with an NRI of 0.376 (*P* < 0.001) and an IDI of 0.025 (*P* < 0.001) (Supplementary Table 1). These results underscore AIP as a complementary, rather than substitutive, biomarker to existing prognostic models, supporting its role as an independent indicator of residual mortality risk in CKD.

## 4 Discussion

In this prospective cohort study based on NHANES data, which included 4,403 participants, we identified a significant association between baseline AIP and mortality risk in patients with CKD. This relationship remained robust for both all-cause and cardiovascular mortality, even after extensive adjustment for a wide range of potential confounding factors. The robustness of these findings was further corroborated through restricted cubic spline analysis, subgroup analyses, and sensitivity tests. This discovery broadens the application of AIP as a prognostic marker in patients with CKD. Moreover, we also verified that adding the AIP value to the traditional risk factor model could improve the predictive capacity for all-cause mortality. In brief, this research demonstrates that a reduced AIP value may be a risk factor for poor prognosis in patients with CKD.

The present study demonstrates that higher AIP quartiles are significantly associated with increased mortality risk in patients with CKD compared to those in the lowest quartile. While investigations into the specific relationship between AIP and CKD remain limited, the association between dyslipidemia and CKD has been widely established (25, 26). However, existing evidence is not entirely consistent. For example, You et al. reported a significant correlation between elevated AIP levels and an increased risk of CKD (27). Similarly, other studies have linked higher AIP levels to increased mortality among patients with diabetes, findings that are in agreement with our results (28).

In contrast, a study conducted in dialysis patients revealed a U-shaped relationship between AIP levels and all-cause mortality, with both the lowest quintile (≤ 0.20) and the highest quintile (≥ 0.71) independently associated with increased mortality risk (29). The pathophysiological mechanisms underlying this observation are incompletely understood but may involve the unique pathophysiological characteristics of dialysis patients, who are typically in the late stages of kidney disease and experience profound metabolic and physiological derangements. Patients in the lowest AIP quintile may suffer from more severe malnutrition and inflammatory states, contributing to heightened mortality risk. Moreover, the coexistence of severe cardiovascular disease, anemia, and electrolyte imbalances—common in dialysis patients—may confound the prognostic value of AIP.

By contrast, the participants in this study were non-dialysis CKD patients, predominantly in the early to middle stages of the disease, with relatively fewer pathological and comorbid complications. This distinction highlights the potential modulatory role of CKD stage and comorbid conditions in the relationship between AIP and patient outcomes. These findings underscore the need for future research to elucidate the differential impact of AIP across various CKD stages and clinical contexts.

Although the precise mechanisms underlying the increased risk of CKD-related mortality associated with AIP remain incompletely understood, several potential explanations exist. Firstly, as a key indicator of lipid metabolism dysregulation, AIP reflects the ratio of TG to HDL-C, which plays a pivotal role in the progression of CKD. Elevated TG levels promote the formation of small, dense low-density lipoprotein cholesterol (LDL-C) particles, which are more prone to oxidative modification (30, 31), thereby enhancing the risk of atherosclerosis. In patients with CKD, impaired renal function disrupts normal lipid metabolism, and elevated AIP further exacerbates this dysregulation. Oxidized LDL-C particles are engulfed by macrophages, transforming into foam cells that accumulate in the subendothelial space, ultimately contributing to the formation of atherosclerotic plaques. As these plaques grow and become unstable, vascular lumen narrowing and blood flow obstruction increase, heightening the risk of cardiovascular events such as myocardial infarction and stroke, directly leading to an increased mortality rate from CVD. Secondly, insulin resistance (IR), which is closely associated with elevated AIP and commonly observed in CKD patients (32, 33), further complicates this relationship. Elevated AIP can impair insulin signaling pathways, reducing cellular sensitivity to insulin and triggering IR. In an IR state, the body compensates by secreting more insulin to maintain glucose homeostasis, resulting in hyperinsulinemia. This condition activates the sympathetic nervous system, inducing vasoconstriction and elevated blood pressure, while also stimulating vascular smooth muscle cell proliferation and migration, accelerating the atherosclerotic process, and increasing the risk of CVD (34–36). Moreover, IR disrupts renal hemodynamics, raising intraglomerular pressure and exacerbating renal function decline, thereby creating a vicious cycle (37) that ultimately elevates both all-cause and CVD mortality rates.

Furthermore, inflammatory responses play a crucial role in AIP-mediated adverse outcomes in CKD patients. Table 1 of this study demonstrates that inflammatory markers, including white blood cell count (WBC), C-reactive protein (CRP), and systemic immune-inflammation index (SII), are significantly elevated in the higher AIP quartiles (Q2-Q4) compared to the Q1 group. Chronic inflammation is a well-established feature of CKD (38, 39), and elevated AIP may further activate immune cells such as monocytes and macrophages. These cells release a multitude of pro-inflammatory cytokines, including tumor necrosis factor-alpha (TNF-α) and interleukin-6 (IL-6), thereby triggering systemic inflammation. Inflammatory cytokines can directly damage endothelial cells, compromising vascular integrity and function, which promotes thrombus formation. Additionally, these cytokines stimulate the proliferation of smooth muscle cells and extracellular matrix production, leading to thickening and stiffening of the vascular wall, worsening atherosclerosis, and increasing the likelihood of CVD events, thereby raising mortality rates. Locally, inflammation exacerbates damage to the glomeruli and renal tubules, accelerating renal function deterioration (40), thereby contributing to an increased risk of all-cause mortality. Furthermore, endothelial dysfunction represents a critical link in the effect of AIP on mortality in CKD patients. The dyslipidemia and inflammatory responses induced by AIP collaborate to impair endothelial cell function (41). A reduction in vasodilators such as nitric oxide (NO) and an increase in vasoconstrictors like endothelin-1 (ET-1) result in impaired vascular dilation and enhanced constriction, creating hemodynamic abnormalities (42). Endothelial dysfunction also facilitates the deposition of lipids on the vascular wall, promoting the progression of atherosclerosis and elevating the risk of CVD events, leading to increased CVD mortality (43). Moreover, endothelial dysfunction in the renal microvasculature can compromise glomerular filtration and tubular reabsorption, accelerating renal function decline and indirectly increasing the risk of all-cause mortality (44).

Additionally, the AIP-related lipid metabolism dysregulation in CKD involves complex molecular mechanisms that warrant further exploration. Emerging evidence suggests that elevated AIP reflects phospholipid dysregulation in CKD, driven by phospholipase A_2_(PLA_2_)-mediated generation of proinflammatory lysophospholipids such as lysophosphatidylcholine (LysoPC) and lysophosphatidic acid (LysoPA) (45). These metabolites not only promote endothelial dysfunction but also accelerate foam cell formation, representing critical steps in atherosclerosis pathogenesis. Furthermore, vitamin D deficiency exacerbates lipid accumulation by impairing mitochondrial fatty acid oxidation (FAO) in renal tubular cells and macrophages, thereby elevating AIP levels and contributing to systemic inflammation (46). Conversely, Klotho, a renal protective protein, inversely correlates with AIP through antioxidative and anti-inflammatory pathways, mitigating fibrosis and oxidative stress in CKD (47). Another key player is adipose triglyceride lipase (ATGL), which maintains FAO and mitochondrial function to reduce lipotoxicity and fibrosis; its deficiency promotes lipid droplet accumulation and inflammatory cytokine release (48). Collectively, these findings highlight PLA_2_hyperactivity, vitamin D deficiency, Klotho loss, and ATGL dysfunction as pivotal determinants of AIP elevation in CKD. Targeting these pathways may offer novel therapeutic strategies to mitigate cardiovascular complications in this population.

In summary, AIP contributes to an increased risk of both all-cause and cardiovascular mortality in CKD patients through multiple mechanisms, including lipid metabolism disturbances, induction of insulin resistance, activation of inflammatory responses, and endothelial dysfunction. These pathways are interrelated, creating a complex pathological network that drives CKD progression. This further underscores the clinical significance of AIP as a prognostic marker in CKD, offering potential targets for future comprehensive treatment strategies aimed at reducing AIP levels and mitigating its adverse effects, ultimately improving long-term survival outcomes for CKD patients.

The interaction analysis in this study revealed significant interactions between gender, BMI, and DM in their effect on all-cause mortality, particularly among women and individuals with higher BMI, where these factors exhibited a more pronounced influence on all-cause mortality. Surprisingly, the predictive value of AIP for all-cause mortality was not statistically significant in the diabetic group when compared to the non-diabetic group. This may be attributed, in part, to the prevalent insulin resistance observed in diabetic patients, which could alter their lipid profile and thus impair AIP’s ability to adequately reflect lipid abnormalities in this population. As a result, the relationship between AIP and all-cause mortality may be attenuated in diabetic individuals. Furthermore, diabetic patients often undergo more intensive medical interventions, including antidiabetic medications, statins, and other cardiovascular protective measures, which may lower AIP levels, thereby diminishing its prognostic value. Additionally, compared to diabetic individuals, the non-diabetic population is less affected by factors such as insulin resistance and multiple comorbidities. AIP changes in this group are more likely to be driven by traditional lipid metabolism abnormalities (e.g., hypertriglyceridemia, low HDL-C levels), which are more directly and clearly associated with all-cause mortality. Thus, the effect of AIP on all-cause mortality may be more readily apparent in the non-diabetic cohort. Finally, other potential mechanisms warrant further investigation. Notably, a separate study has indicated that although the predictive value of AIP in diabetic patients may be influenced by other factors, it retains incremental prognostic value in individuals with acute coronary syndrome and can serve as a vital tool for risk stratification and prognosis assessment (49), which aligns with the subgroup analysis results of our current study regarding CVD mortality.

The AIP is derived from routine lipid measurements, specifically TG and HDL-C, and requires no additional cost or specialized testing. Its ease of calculation offers excellent clinical applicability, enabling effective implementation even in environments with relatively limited medical resources. Compared to traditional methods that measure TG or HDL-C separately, AIP integrates two key indicators of lipid metabolism. By quantifying the dynamic balance between TG and protective HDL-C, this index provides a more precise assessment of residual cardiovascular risk in patients with CKD. Particularly in the CKD population with LDL-C within the reference range but exhibiting lipid metabolism disorders, AIP demonstrates superior risk prediction capabilities compared to traditional lipid parameters. Furthermore, while traditional markers for predicting adverse outcomes in CKD, such as the urine albumin-to-creatinine ratio (UACR) and eGFR, have been well validated, these parameters primarily reflect renal structural damage and functional decline. In contrast, AIP reflects systemic lipid metabolism disorders, representing a unique pathophysiological pathway associated with cardiovascular complications and inflammation in CKD, which cannot be fully captured by UACR or eGFR alone. Consequently, AIP serves as a complementary assessment tool for the pathological processes not addressed by traditional renal function markers, thereby enhancing the overall prognostic assessment for patients with CKD.

Our study presents several strengths. First, the sample exhibits strong representativeness. The NHANES database employs a complex weighting design, and this study utilized data from 1999 to 2018, encompassing a broad spectrum of adult CKD patients in the United States. This robust sample is well-suited to reflect the overall population characteristics, thereby enhancing the generalizability of the findings. Second, the measurement of variables is precise. AIP and a range of covariates, including demographic information, laboratory markers, and chronic comorbidities, were measured using standardized methodologies. Data collection was performed by trained professionals following standardized protocols, and blood samples were analyzed in the same laboratory, minimizing bias and ensuring high data quality, thereby strengthening the credibility of the findings. Third, the analytical methods employed were comprehensive. Various adjustments for potential confounders, along with stratified analyses, were utilized to ensure the reliability and consistency of the results.

However, there are certain limitations in our study. First, It is crucial to emphasize that this study represents an observational cohort design, and as such, it is not possible to ascertain a causal relationship between AIP and mortality outcomes among CKD patients. Despite our adjustment for an extensive array of potential confounders, residual confounding factors, including unmeasured lifestyle variables or genetic influences, might still affect the observed associations. Further interventional studies are warranted to investigate whether modulating AIP levels can directly enhance clinical outcomes in CKD patients. Second, Our analysis primarily utilized baseline measurements of covariates, including AIP, comorbidities, and medication use. While this approach is common in cohort studies, it does not account for potential dynamic changes during follow-up, such as the onset of new diabetes, decline in eGFR, or initiation of lipid-lowering therapies. These time-varying factors may confound the observed associations, particularly in progressive diseases like CKD, where risk profiles evolve over time. This limitation is inherent to datasets like NHANES, which lack repeated measurements of covariates during follow-up. Future studies with longitudinal biomarker and comorbidity data could refine these findings. Third, while NHANES mortality linkage via the NDI provides robust follow-up data, participants are not actively monitored over time, and certain subgroups (e.g., transient populations) may be underrepresented. However, NHANES’s rigorous sampling methodology and weighting adjustments help ensure generalizability to the U.S. non-institutionalized CKD population. Fourth, while our analysis of CVD mortality accounted for non-CVD deaths using the Fine-Gray model, it is important to note that all-cause mortality inherently accounts for competing risks (e.g., non-CVD deaths). Future studies should explore other competing outcomes (e.g., infection, malignancy) in larger CKD cohorts to comprehensively evaluate the impact of AIP on mortality. Finally, a notable limitation of this study is the absence of external validation in an independent CKD cohort. While NHANES provides robust, population-representative data, replication in diverse cohorts (e.g., Asian or European populations) is essential to confirm the generalizability of our findings. Future studies should prioritize validation in independent datasets to further solidify the prognostic role of AIP in CKD. Notwithstanding this limitation, our study benefits from a large, ethnically diverse sample and long follow-up period, providing robust evidence for the association between AIP and mortality in U.S. CKD patients. The consistency of results across subgroup analyses further supports the reliability of our findings.

## 5 Conclusion

Based on data from the U.S. NHANES spanning from 1999 to 2018, this study reveals a significant association between AIP and the risk of all-cause mortality as well as CVD mortality in patients with CKD. A significant positive correlation was identified between AIP and mortality risk, which remained robust whether AIP was analyzed as a continuous variable or categorized into quartiles. Incorporating AIP into clinical risk stratification models may enhance the identification of CKD patients at elevated risk for mortality, enabling targeted interventions such as lipid-lowering therapies and lifestyle modifications. However, critical knowledge gaps persist, including the absence of validated AIP-specific therapeutic targets and randomized controlled trials (RCTs) assessing the efficacy of AIP-driven treatment protocols. Prospective validation studies are warranted to determine optimal AIP thresholds and evaluate the impact of AIP-lowering interventions on clinical outcomes. For patients with elevated AIP of Plasma, clinicians should prioritize lifestyle modifications, such as adopting a Mediterranean diet and engaging in aerobic exercise (50). Furthermore, pharmacological interventions, including fibrates and omega-3 fatty acids, should be considered to lower triglyceride levels (51). Intensified monitoring for cardiovascular events and potential decline in renal function is also recommended.

In conclusion, our study demonstrates that AIP is associated with adverse outcomes in CKD and may be considered a potential biomarker for risk assessment. However, the observational nature of our analysis precludes causal inferences. Future research should focus on mechanistic studies elucidating AIP’s pathophysiological role and interventional trials testing AIP-guided therapy. These efforts may ultimately refine prognostic models and inform precision medicine approaches in CKD management.

## Data Availability

Publicly available datasets were analyzed in this study. This data can be found at: For details on survey design and data acquisition, please refer to the NHANES website at https://www.cdc.gov/nchs/nhanes/.
