# Supplementary material for: Association between the atherogenic index of plasma and mortality in the chronic kidney disease population: evidence from NHANES
Source: Front Med (Lausanne). 2025 May 30;12:1575657. doi: 10.3389/fmed.2025.1575657 (PMC12163239; doi:10.3389/fmed.2025.1575657)
Supplement: Supplementary file 1 [file Table_1.doc]

**Table S1.** C-index, NRI, and IDI for AIP predictive value of all-cause death

| Variable | C-statistic | | NRI | | IDI | |
| --- | --- | --- | --- | --- | --- | --- |
|  | Estimate (95%CI) | *P* value | Estimate (95%CI) | *P* value | Estimate (95%CI) | *P* value |
| AIP | 0.713 (0.571-0.855) | 0.007 | - | | - | |
| Traditional factors mode | 0.811 (0.793-0.829) | <0.001 | Reference | | Reference | |
| Traditional factors + AIP model | 0.823 (0.806-0.841) | <0.001 | 0.376 (0.293-0.459) | <0.001 | 0.025 (0.019-0.031) | <0.001 |

Traditional factors = gender, age, smoking status, drinking status, education levels, marriage status,FPIR, hypertension, diabetes, CVD history, Lipid lowering drugs history, CRP, SII, LDL-c, eGFR.

IDI = integrated discrimination improvement, NRI = net reclassification improvement.
