# Supplementary material for: Association between the atherogenic index of plasma and mortality in the chronic kidney disease population: evidence from NHANES
Source: Front Med (Lausanne). 2025 May 30;12:1575657. doi: 10.3389/fmed.2025.1575657 (PMC12163239; doi:10.3389/fmed.2025.1575657)

Figure S1. (A) Cumulative incidence of cancer (CA)-related mortality analyzed using Fine-Gray competing risk models, accounting for non-CA deaths (e.g., cardiovascular, infection) as competing events. Participants were stratified by AIP quartiles (Q1–Q4). The Gray's test confirmed no significant differences in CA-related mortality risk across AIP quartiles (*P* = 0.37). **(B)** Cumulative incidence of infection-related mortality analyzed using Fine-Gray competing risk models, accounting for non-infection deaths as competing events. Gray's test showed no significant differences in infection-related mortality risk across AIP quartiles (*P* = 0.61). Q1: AIP ≤ -0.19; Q2: -0.19 to 0.02; Q3: 0.02 to 0.24; Q4: >0.24. Shaded areas represent 95% confidence intervals. Data were weighted to represent the U.S. non-institutionalized population.

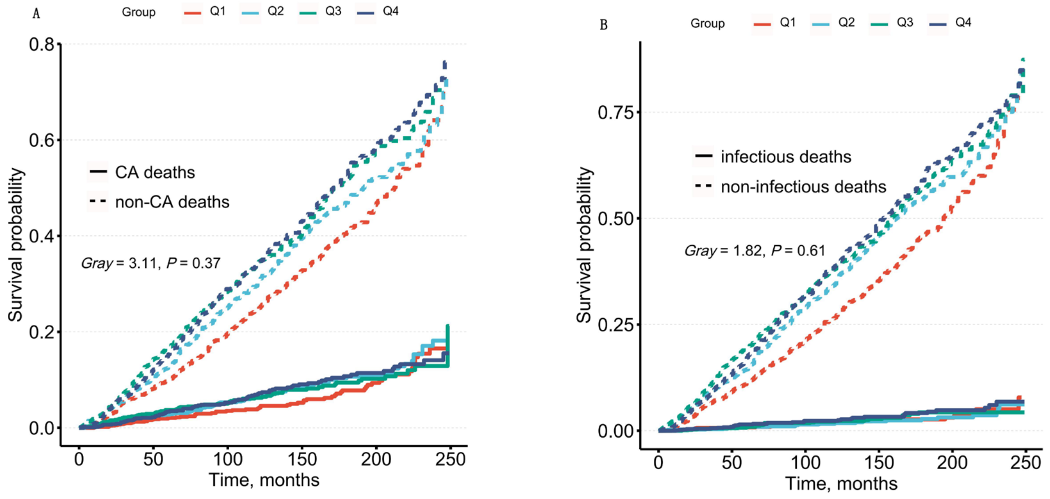

Supplement: Supplementary file 2 [file Image_1.pdf]
